# Role of landslides on the volume balance of the Nepal 2015 earthquake sequence

**DOI:** 10.1038/s41598-021-83037-y

**Published:** 2021-02-09

**Authors:** A. Valagussa, P. Frattini, E. Valbuzzi, G. B. Crosta

**Affiliations:** Department of Earth and Environmental Sciences, University of Milano-Bicocca, Milan, Italy

**Keywords:** Natural hazards, Geology, Geomorphology

## Abstract

The 7.8 M_w_ earthquake that struck Nepal on April 25th, 2015 triggered over 21,000 landslides over an area of more than 25,000 km^2^. These landslides contributed to mass wasting, partially compensating the tectonic uplift by the earthquake. In this paper we quantify the volume balance resulting from the 2015 earthquake uplift (or subsidence) and landslide erosion. Starting from a new complete earthquake-induced landslide inventory, we calculated landslide volume by adopting different strategies for low-mobility and high-mobility landslides, considering also the potential supply of sediments to the drainage network. The results show that the contribution of earthquake-induced landslides to erosion is about one order of magnitude smaller than the vertical coseismic displacement. We found landslide volume values, due to the 2015 Nepal earthquake, ranging between 251 (− 15/ + 16) Mm^3^ up to 1503 (− 183/ + 210) Mm^3^ based on the adopted method, and a volume due to coseismic vertical displacement of 2134 (± 1269) Mm^3^ for the whole area. The volume balance of the 2015 Nepal earthquake is strongly dominated by tectonic displacement. We show that these estimates depend on several uncertainties. We identified and quantified uncertainties related to: (1) the choice of empirical volume-area scaling relationships and their parameters; (2) the completeness and quality of landslide inventory through comparison with available inventories; (3) the approach adopted for the assessment of elongated landslide volume; (4) the InSAR displacement data.

## Introduction

Shallow earthquakes are the most important driver of rock uplift in mountain ranges, through repeated vertical displacements^1^. Moreover, earthquakes are a primary trigger of landslides^2^ that may have a relevant role in the mountain belt evolution^3–6^ by causing intense erosion. The point and area density^7,8^ and the size (i.e. area and volume) of slope failures were found to be potentially proportional to specific seismic parameters, such as the peak ground acceleration and the earthquake magnitude^9–12^. Therefore, larger earthquakes are commonly associated to a larger erosional impact^2^.

The net volume balance between seismically-induced rock uplift and landslide erosion may differ between earthquakes^13^. In some cases, the tectonic uplift or subsidence was higher than topographic lowering due to earthquake-induced landslides (e.g., 1999 Chi‐Chi earthquake^14^; 2010 El Cucapah Mayor earthquake^15^). In other cases, such as the 2008 Wenchuan earthquake, the landslide mass wasting matched the seismic uplift^6,16^. Starting from these observations, Li et al.^16^ suggested that earthquakes larger than a critical magnitude would allow erosion to compensate the rock uplift. Based on a model of earthquake‐triggered landslides and an analytical solution of coseismic surface displacement, Marc et al.^13^ suggested that earthquakes with a net erosive effect are not those with a large magnitude, above M_w_ 8, but rather those with intermediate magnitudes between M_w_ 6.3 and 7.3. On the other hand, earthquakes with both smaller and larger magnitude (M_w_ < 6, M_w_ > 7.3) have a net positive volume balance. As far as we know, an estimate of the net volume balance associated to the 2015 Nepal earthquake is still missing, although some authors already tried to estimate the volume of landslides occurred during the earthquake^17,18^. These volumes are consistent with the landslide volume-earthquake magnitude relationships available in the literature^2,9,10^. However, due to the large rock uplift that occurred during the earthquake^19–21^, our first hypothesis is that the overall contribution of landslide erosion to the volume balance of the 2015 Nepal earthquake is small with respect to tectonic uplift, differently from what was observed for other earthquakes (e.g. Li et al.^16^). This is important because it reveals how single large earthquakes may significantly contribute to the overall surface uplift of the Nepal Himalayan belt. A second hypothesis is that the uncertainty related to either the landslide volume or the vertical coseismic displacement is very high, of the same order of magnitude of the estimated values.

## The 2015 Nepal earthquake landslide inventory

**Figure 1:**
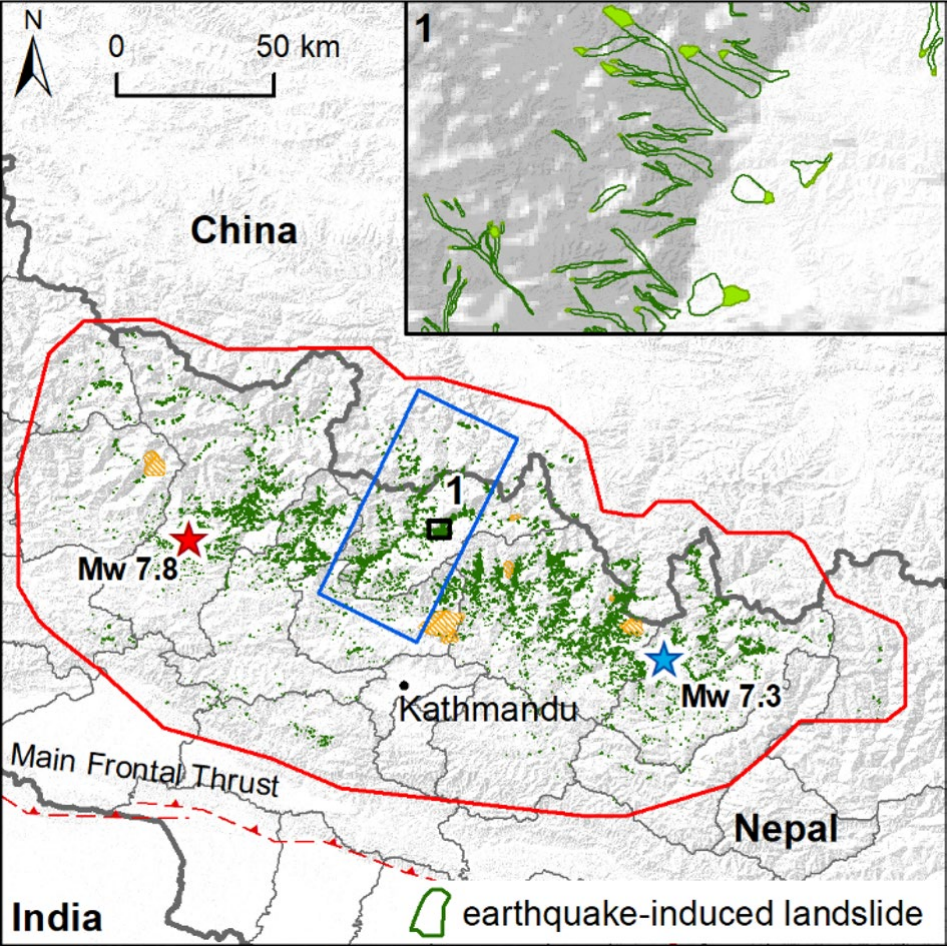
Earthquake-induced landslides inventory, with the 21,151 landslides mapped as a polygons. The bold grey line is the boundary of Nepal, while the thin grey lines are the district boundaries in Nepal. The blue rectangle identifies the area (2230 km^2^) where the scar area was identified and mapped as an independent feature for 1500 out of 2258 landslides. The red and blue stars localize the main foreshock and the main aftershock, respectively. The inset 1 shows an example of landslides mapped as polygons in the inventory, with the distinction of scar (filled) and runout (outline) areas. The red line indicates the study area. Orange areas indicate unmapped areas due to clouds cover or low quality images. (The map was generated by using ArcGIS 10.3.1, http://www.esri.com/).

On April 25th, 2015, an earthquake (M_w_ 7.8) occurred 80 km to the northwest of Kathmandu. The earthquake, known as the Gorkha earthquake^20,22–24^, is the result of faulting on the main thrust plane (Main Himalayan Frontal Thrust, MFT) between the subducting Indian plate to the south and the overriding Eurasian plate to the north. The largest aftershock (M_w_ 7.3) occurred on May 12, 2015, 80 km to the east of Kathmandu. The area affected by the earthquake extends between Nepal and China (Fig. 1).

Immediately after the event, various research groups mapped the co-seismic landslides^25,26^ as points or lines. In addition, Zhang et al. ^27^, and Lacroix^28^ mapped landslides as polygons for small areas. Gnyawali and Adhikari^29^ mapped 17,628 landslides as polygons based on high-resolution images available in Google Earth^29^ (Supplementary Figure S1). Martha et al.^30^ developed an inventory with 15,551 landslides using high-resolution satellite data. Roback et al.^17^ developed a co-seismic landslide inventory where source and runout areas are differentiated. The inventory contains 24,915 landslides and it was realized by using high-resolution pre- and post-event satellite imagery (Supplementary Figure S1). Another inventory (47,200 landslides) was realized by Xu^31^ based on visual interpretation of pre- and post-earthquake high-resolution optical satellite images and field reconnaissance. Finally, Valagussa et al.^32^ developed an inventory of 4300 landslides, mainly located in the central part of the area affected by the earthquake. This inventory was subsequently completed and used for all the analyses presented in this study. The inventory was prepared, at a 1:2000 scale through manual mapping of landslide polygons on available multi-temporal images, and helicopter-based videos. The inventory covers an area of about 25,000 km^2^ and contains 21,151 landslides (Fig. 1; Supplementary Figure S1). The mapped landslides were classified as debris flows, shallow translational slides, rockfalls, and in the upper sector of the mountain belt, rock-ice avalanches and rock-ice falls.

Manual mapping of landslides avoids or minimizes common problems recognized in landslide inventories such as amalgamation^11,33^. Manual mapping allows accurate description of the shape and the size of each landslide, thus allowing a reliable analysis of denudation rate, sediment yield and size frequency density at a regional scale.

On the other hand, some problems may exist with: the exact positioning of landslides, due to inaccuracies in the georeferencing of the Google Earth imagery, the image rectification, the lighting, the vegetation cover, and the steep slope of the topography^34^. While the first issue does not affect the study of volume balance, the other issues may introduce uncertainties in the landslide footprint and therefore in the estimation of the landslide volumes. Where available, helicopter-based videos were used to resolve some of the mentioned issues. In addition, field checks of a small part of the landslide inventory were carried out in October 2015 in the Rasuwa and Sindhupalchok districts. A subsequent validation was performed based on the photos taken in the field. Another issue with manual mapping is the consistency of interpretation and mapping among different interpreters. For this reason, the inventory was entirely mapped by a single geomorphologist, crosschecked with other expert mappers and by using available satellite images and helicopter-based video.

## Landslide scar area

The mapping of the landslide scar is important for volume estimation^35^. Due to the variable quality of the images used for landslide mapping, it was extremely difficult to consistently separate the scarp and the runout areas throughout the study area. Hence, this separation was completed for a subset of well visible 1500 landslides of different type (Fig. 1) for which the scar ratio (SR, i.e. the slope of the best-fitting linear regression between scar area and total landslide area) and the aspect ratio (AR, i.e. the ratio between the landslide length and width) were calculated. Since it was expected the SR to be a function of the landslide shape and elongation, landslides were classified into six AR classes for which different relationships between the total landslide area and the main scar area were attained.

**Figure 2:**
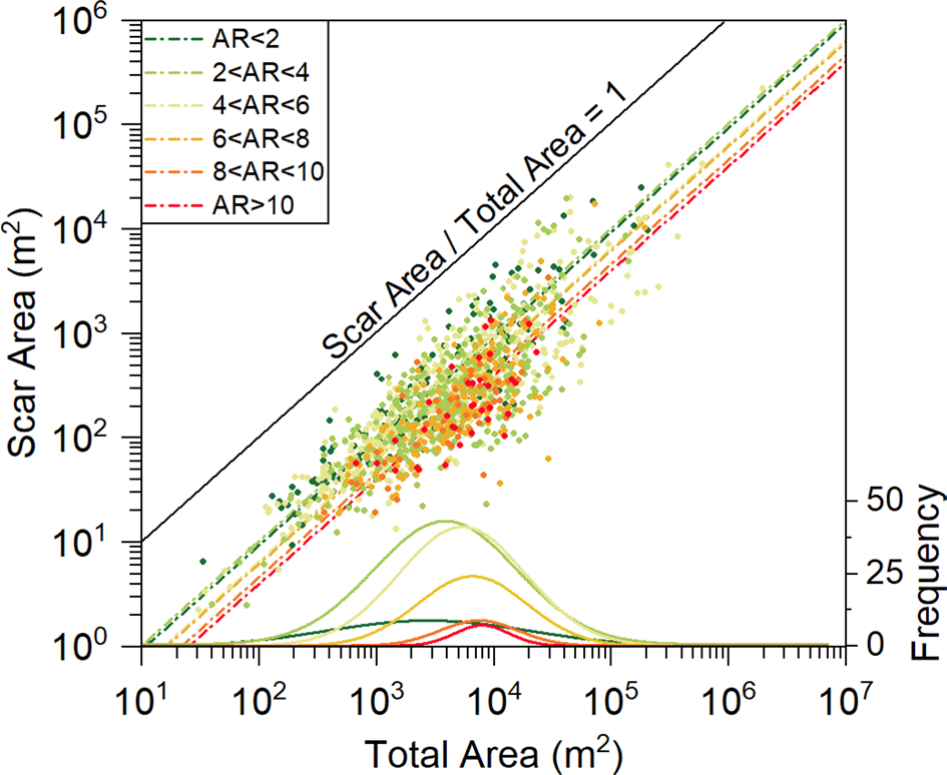
Scatterplot showing the relationship between the scar area and the total landslide area as obtained for a subsample of 1500 landslides from the inventory (Fig. 1). The points are coloured according to the aspect ratio (AR). The linear fitting functions (Table 2) and the landslides size frequency distribution for each AR class are also reported.

As expected, the modelled SR declines with the degree of elongation: the more elongated the landslide, the smaller the ratio (Fig. 2 and Table 1). The size frequency distribution of landslides belonging to the different aspect ratio (AR) classes shows a shift toward larger landslides for higher AR ratios, together with an overall reduction in frequency (Fig. 2).

**Table 1:** Linear functions describing the relationships between the scar area (A_s_) and the total landslide area (A), for six different classes of the aspect ratio (AR).

AR	Fitting function	Landslide scar area (As, %)
< 2	A_s_ = 0.093A	9.3
2–4	A_s_ = 0.102A	10.2
4–6	A_s_ = 0.065A	6.5
6–8	A_s_ = 0.062A	6.2
8–10	A_s_ = 0.046A	4.6
> 10	A_s_ = 0.040A	4.0

The percentages quantify the amount of landslide scar area with respect to the total landslide area (see Supplementary Table S1).

## Landslide volume and erosion

To estimate the landslide volume removed by the earthquake, the study area was divided in 235 lattice cells, with a dimension of 10 × 10 km. For each cell, the landslide volume was calculated with three approaches to analyse the source of uncertainty in landslide volume assessment: (1) V_ALL in which the volume was defined for all the landslides by using two volume/area relationships R1 (global relationship for all landslides) and R2 (relationship for Himalayan landslides)^36^; V_AR, for which the inventory was separated into elongated (AR > 3) and non-elongated (AR ≤ 3) landslides. For non-elongated landslides the same approach as V_ALL was applied. For elongated landslides, the volume was calculated by applying R1 and R2 to the scar area only, while the contribution of erosion along the runout path was calculated by assuming an erosion thickness of 0.5, 1 and 3 m; (2) V_AR_river, in which the volume was defined as for V_AR, but only for landslides that reached the channel network (see “Methods” for details).

The volumes calculated with this three approaches (V_ALL, V_AR, and V_AR_river) are significantly different, and different for the same approach with the two adopted formulas (R1, global relationship for all landslides and R2, relationship for Himalayan landslides, see “Methods”). In general, the mean erosion values obtained with R2 are much higher than the values obtained with R1. For V_AR_river the differences between the two formulas are smaller due to the reduced number of landslides on which the analysis is conducted (23% of the entire inventory) and their covered area.

**Figure 3:**
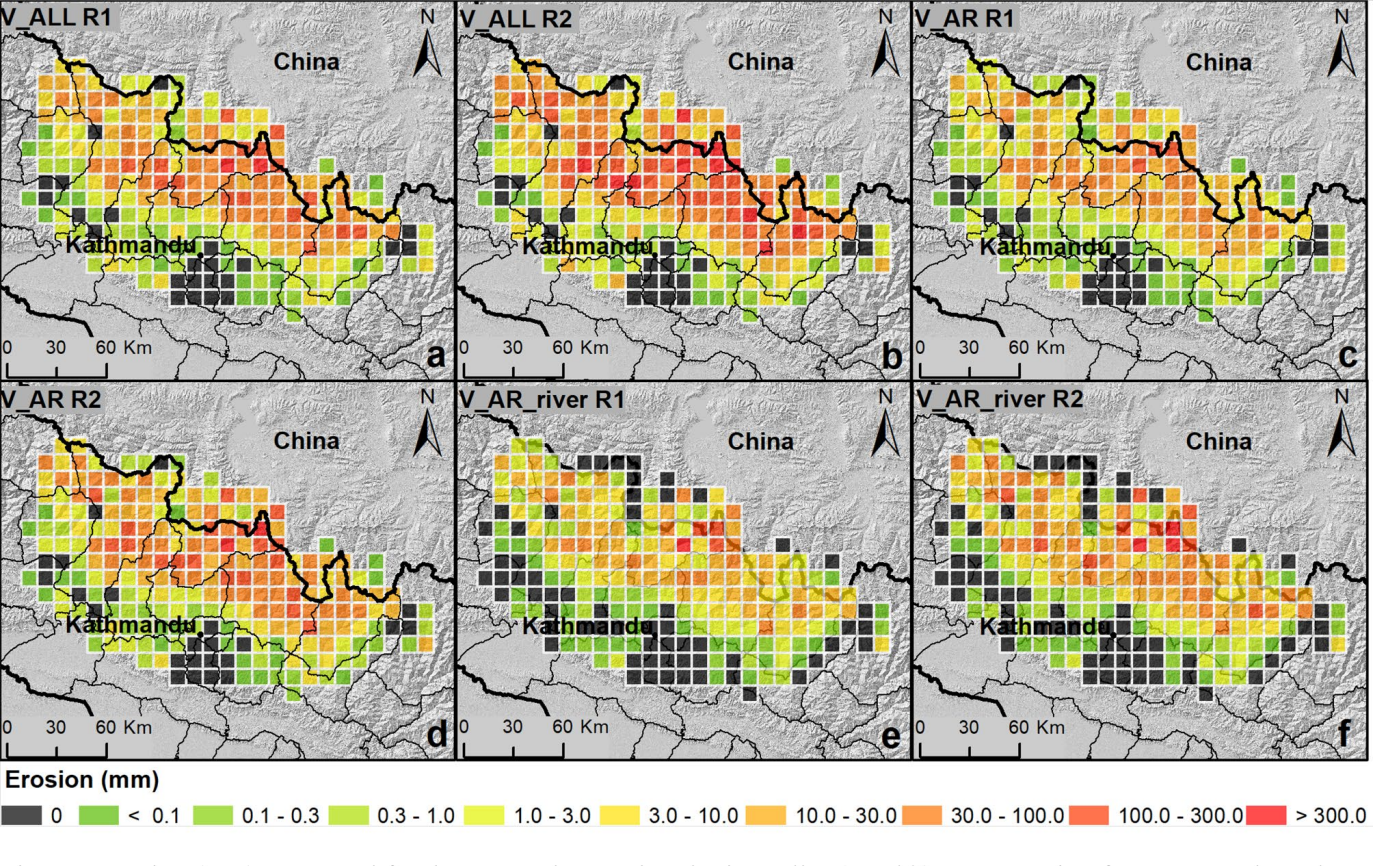
erosion (E_cell_) computed for the 235, 10 km x10km, lattice cells. (**a**,**b**) Mean erosion for V_ALL: the volume is calculated from the entire landslide area with R1 (global relationship for all landslides) and R2 (relationship for Himalayan landslides), respectively (see Supplementary Table S2). (**c**,**d**) Mean erosion for V_AR. The landslides volume was calculated with R1 and R2, respectively; (**e**,**f**) mean erosion for landslides that overlap the river network, V_AR_river, again with R1 and R2, respectively. For the scar area both R1 and R2 were applied. For V_AR and V_AR_river, the volume eroded from the runout area is computed assuming an erosion depth ranging between 0.5 and 3 m. Black lines indicate the districts in Nepal. (The maps were generated by using ArcGIS 10.3.1, http://www.esri.com/).

By considering the total landslide area (V_ALL), the mean erosion averaged over the 10 × 10 km lattice cells is about 30 and 70 mm for R1 and R2, respectively. The erosion is over 50 mm for 37 (R1) and 71 (R2) lattice cells located along the sub-Himalayan range between the epicentres of mainshock and main aftershock (Fig. 3a,b, orange/red lattice cells). For the whole area, the total eroded volume amount to 628 (− 39/ + 42) Mm^3^ and 1503 (− 183/ + 210) Mm^3^ by R1 and R2, respectively. The uncertainty on the volume estimation was assessed by mean values ± 16th and 84th percentiles of 10,000 Monte Carlo samplings for α and γ scaling parameters.

The mean erosion values over the 10 × 10 km lattice cells range between 26.7 (− 1.7/ + 1.8) mm to 64.0 (− 7.8/ + 8.9) mm for R1 and R2, respectively.

By adopting the V_AR approach, a significant decrease in mean erosion is observed. The mean erosion over the 10 × 10 km lattice cells is about 15 and 30 mm, with R1 and R2, respectively. The number of lattice cells with an erosion higher than 50 mm decreases to 21 (R1) and 43 (R2), and the total eroded volume is almost halved amounting to about 324 (− 15/ + 16) Mm^3^ and 660 (− 70/ + 81) Mm^3^ by R1 and R2, respectively. The mean erosion values over the 10 × 10 km lattice cells range between 13.8 (− 0.6/ + 0.7) mm to 28.1 (− 3.0/ + 3.4) mm for R1 and R2, respectively (Fig. 3c,d).

By considering only landslides supplying sediment to the channel network (V_AR_river), the total eroded volume amounts to about 251 (− 15/ + 16) Mm^3^ (R1) and 582 (− 72/ + 83) Mm^3^ (R2). The mean erosion over the 10 × 10 km lattice cells range between 10.7 (− 0.6/ + 0.7) mm to 24.8 (− 3.1/ + 3.5) mm for R1 and R2, respectively (Fig. 3e,f). The uncertainty on erosion values is propagated from the uncertainty on landslide volume.

**Figure 4:**
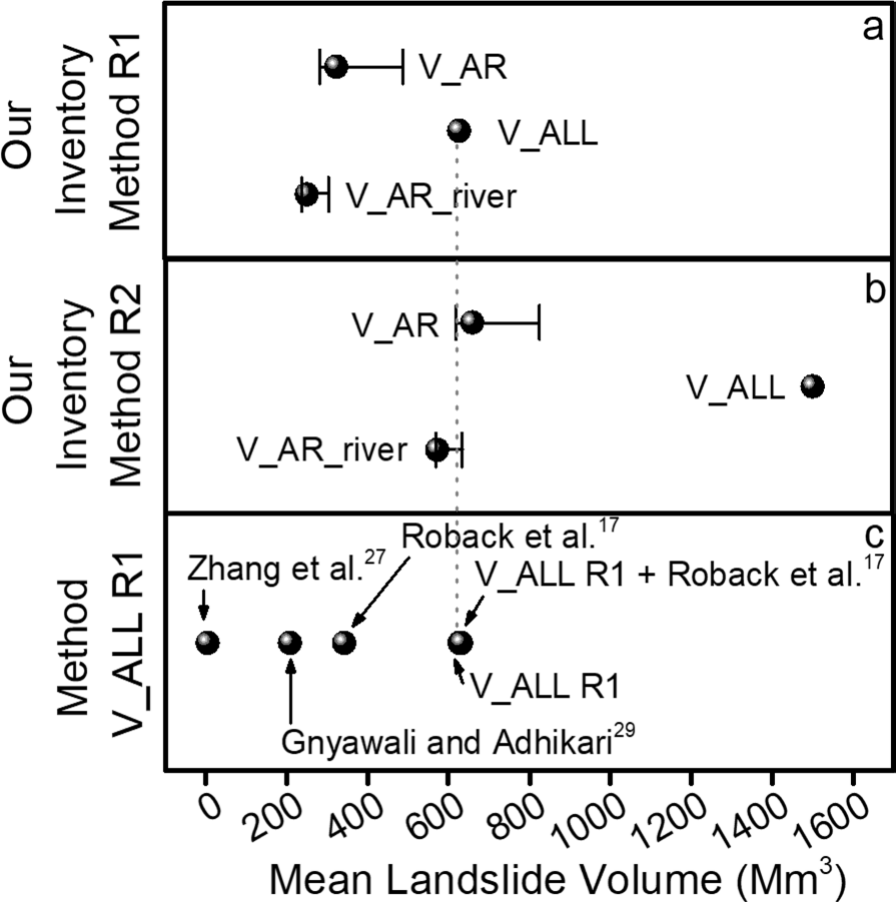
(**a**) Mean landslide volume (Mm^3^) for the three methods based on R1. (**b**) Mean landslide volume (Mm^3^) for the three methods based on R2. In (**a**,**b**) horizontal bars indicate the range of variation due to a change in the soil thickness eroded along the runout area (0.5 and 3 m). (**c**) Mean landslide volume (Mm^3^) for the earthquake-induced landslide inventories in Nepal: Roback et al.^17^; Gnyawali and Adhikari^29^; Zhang et al.^27^ and the integration in our inventory of landslides by Roback et al.^17^ located in unmapped areas (V_ALL R1 + Roback et al.^17^).

The differences between the proposed methods for the assessment of the landslide volumes are clearly shown by Fig. 4a. It is possible to observe that the selection of different equations and approaches gives a range of variation between 251 Mm^3^ and 1503 Mm^3^. Change in the values of the considered soil thickness (0.5 and 3 m) along the runout area does not return significant change in the calculated volume (black line in Fig. 4a,b). In addition Fig. 4c underlines the importance of the input data involved in the analysis. The total landslide volumes calculated by applying V_ALL R1 method for the three landslide inventories available online are: 6 Mm^3^ for Zhang et al.^27^; 209 Mm^3^ for Gnyawali and Adhikari^29^; 343 Mm^3^ for Roback et al.^17^. The integration of landslides by Roback et al.^17^ in our inventory only for areas that we have not mapped due to the presence of clouds or high-distorted images leads to only a small increase in estimated landslide volume, from 628 Mm^3^ (V_ALL R1) to 631 Mm^3^ (V_ALL R1 + Roback et al.^17^ in Fig. 4c).

## Vertical coseismic displacement

The April 2015 M_w_ 7.8 earthquake occurred in a region with less than optimal seismic and geodetic coverage^19^. For this reason, the vertical displacement was analysed by using satellite InSAR data. Two satellites were operational before and after the earthquake allowing measurements of line-of-sight (LOS) displacement: the Sentinel-1a satellite (operated by the European Space Agency—ESA) and the ALOS-2 satellite (operated by the Japanese Space Agency—JAXA)^19^ which collect C‐band (5.6 cm wavelength) and L-band (23.5 cm wavelength) InSAR observations, respectively (Supplementary Figure S3). In this work, we adopted the ALOS-2 LOS displacement data processed by Lindsey et al. (2015) and corrected to assess the mean vertical coseismic displacement (VCD) for the study area (Fig. 6).

According to Wang and Fialko^37^ the ALOS-2 estimates for the path T048^19^ are affected by a RMSE of 54 mm. When this value is applied over the study area, this is equivalent to an estimated uncertainty of 1269 Mm^3^ on the total coseismic volume^6^.

The uncertainty on this estimate is also shown by the comparison with Sentinel 1 data with GPS data^22^ (see Supplementary Figures S2, S3).

**Figure 5:**
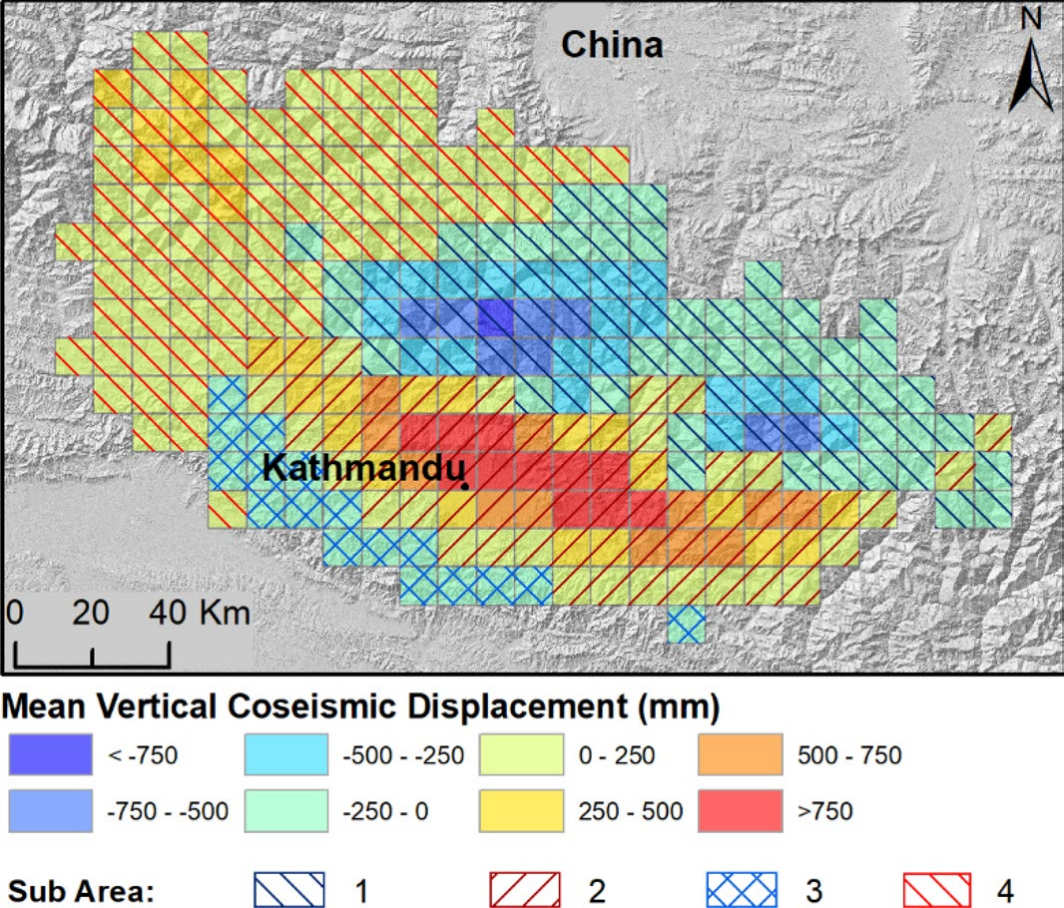
Mean vertical coseismic displacement (VCD, mm) computed in each 10 × 10 km cell of the study area from ALOS-2 InSAR data (Supplementary Figure S4). Negative and positive values indicate ground subsidence and uplift, respectively. Black lines indicate the districts in Nepal. The four sub-areas (1, 2, 3 and 4), identified according to the displacement values, are shown. The vertical coseismic displacement is derived from the LOS displacement developed by Lindsey et al.^19^. (The map was generated by using ArcGIS 10.3.1, http://www.esri.com/).

As found in the literature^19–21^, the mean vertical coseismic displacement allowed us to identify four different sub-areas. Subsidence of up to − 0.9 m (average value − 0.2 m) along the sub-Himalayan and Himalayan sectors N/NE of Kathmandu (sub-area 1 in Fig. 5) was observed roughly between the two main epicentres of the earthquake sequence. This sub-area covers 7300 km^2^. Conversely, rock uplift up to 1.3 m (average value 0.4 m) was observed within an area that extends ESE–WNW from Kathmandu. This zone, defined as sub-area 2 for the subsequent analysis, covers an area of 6700 km^2^ (Fig. 5). A third area south of Kathmandu, with an extent of 1700 km^2^, is characterized by a slight subsidence (sub-area 3, Fig. 5). Finally, a fourth area is located west of Kathmandu. The area extends for 7800 km^2^ and it is characterized by a slight rock uplift with an average value of 0.1 m and a maximum of 0.6 m (sub-area 4, Fig. 5).

Considering the entire area, an average positive vertical coseismic displacement of 91 mm was calculated, corresponding to a total coseismic volume addition to the entire area of 2134 (± 1269) Mm^3^.

## Net volume change

The net volume change was calculated as the difference between the volume due to the coseismic vertical displacement and the volume due to landsliding. For the whole area, a coseismic volume uplift equal to 2134 (± 1269) Mm^3^ was found (Table 2). The net volume change ranging between 1505 (− 1308/ + 1311) Mm^3^ (R1) and 630 (− 1452/ + 1479) Mm^3^ (R2) for the V_ALL method. This value decreases to 1809 (− 1284/ + 1285) Mm^3^ (R1) and 1473 (− 1339/ + 1350) Mm^3^ (R2) for V_AR method and to 1882 (− 1284/ + 1285) Mm^3^ (R1) and 1551 (− 1341/ + 1352) Mm^3^ (R2) for V_AR_river.

**Table 2:** Net volume change (Mm^3^) correlating the coseismic volume change due to the earthquake and the earthquake-induced landslides volume.

	Study area	Sub-area1	Sub-area2	Sub-area3	Sub-area4
**Net volume change (Mm**^**3**^**)**					
ALOS-2 Coseismic volume change (Mm^3^)	2134 (± 1269)	− 1309 (± 394)	2649 (± 362)	− 54 (± 92)	848 (± 421)
V_ALL_R1	1506 (− 1308/ + 1311)	− 1752 (− 422/ + 424)	2598 (± 365)	− 57 (± 92)	716 (− 429/ + 430)
V_ALL_R2	630 (− 1452/ + 1479)	− 2375 (− 525/ + 545)	2531 (− 375/ + 377)	− 59 (± 93)	533 (− 459/ + 464)
V_AR_R1	1809 (− 1284/ + 1285)	− 1521 (− 404/ + 405)	2619 (± 363)	− 55 (± 92)	767 (± 425)
V_AR_R2	1473 (− 1339/ + 1350)	− 1738 (− 439/ + 446)	2591 (− 367/ + 368)	− 56 (± 92)	676 (− 440/ + 443)
V_AR_river_R1	1882 (− 1284/ + 1285)	− 1500 (− 406/ + 407)	2637 (± 363)	− 55 (± 92)	799 (± 424)
V_AR_river_R2	1551 (− 1341/ + 1352)	− 1757 (− 450/ + 459)	2625 (± 365)	− 55 (± 92)	738 (− 434/ + 436)

The data are reported both for the whole study area and for the three sub-areas identified that represent portions of the study area characterized by different uplift and subsidence rates (see Fig. 7). The coseismic volume change from ALOS-2 is reported for comparison in the first line. *R1* global relationship for all landslide types, *R2* relationship for mixed bedrock and soil landslides in the Himalaya^36^. The range of variation is propagated from the uncertainty on the ALOS-2 coseismic volume change and landslide volume.

Considering the subdivision of the study area based on the mean vertical displacement, the largest net surface change is found in sub-area 1. In addition to a coseismic volume change of − 1309 (± 394) Mm^3^ (subsidence) a landslide volume ranging between 442 (− 28/ + 30) Mm^3^ (R1) to 1066 (− 131/ + 151) Mm^3^ (R2) was computed with V_ALL. The landslide volume decreases to 212 (− 10/ + 11) Mm^3^ (R1) and 429 (− 45/ + 52) Mm^3^ (R2) for V_AR, and to 191 (− 12/ + 13) Mm^3^ (R1) and 447 (− 56/ + 65) Mm^3^ (R2) for V_AR_river (Table 2). The sub-area 2 is affected by rock uplift, with a coseismic volume contribution value of 2649 (± 362) Mm^3^, with a landslide volume ranging between 51 (± 3) Mm^3^ (R1) and 118 (− 13/ + 15) Mm^3^ (R2) for V_ALL. This value decreases to 31 (± 1) Mm^3^ (R1) and 59 (− 5/ + 6) Mm^3^ (R2) for V_AR, and to 12 (± 1) Mm^3^ (R1) and 25 (± 3) Mm^3^ (R2) for V_AR_river (Table 2). Sub-area 3 shows a small subsidence coseismic volume equal to − 54 (± 92) Mm^3^ and, due to a limited occurrence of landslides, a slight eroded volume (Table 2). Finally, sub-area 4 shows a coseismic volume due to vertical displacement of 848 (± 421) Mm^3^, partially compensated by a landslide volume ranging between 132 (− 8/ + 9) Mm^3^ (R1) and 315 (− 38/ + 43) Mm^3^ (R2) for V_ALL. With V_AR approach, the landslide volume decreases to 81 (± 4) Mm^3^ (R1) and 172 (− 19/ + 22) Mm^3^ (R2), and to 49 (± 3) Mm^3^ (R1) and 110 (− 13/ + 15) Mm^3^ (R2) for V_AR_river (Table 2).

To evaluate the sensitivity of InSAR data from different satellites, the net volume change for the method V_ALL R1 was calculated also with Sentinel-1 data. For a sub-area in which the two InSAR datasets are overlapping (Supplementary Figures S2, S3), the mean net change values amount to 1417 Mm^3^ and 1841 Mm^3^ for ALOS-2 and Sentinel-1, respectively.

## Discussion

This study allowed quantification of the net change in volume due to the 2015 Nepal earthquake and the triggered landslides.

As stated in the introduction, our first hypothesis was that the contribution of erosion due to earthquake-induced landslides should be small when compared to the amount of coseismic volume change in the study area. The results confirm this hypothesis, showing that the landslide erosion ranges between 12 and 70% of the vertical displacement depending on the adopted approaches. This is different from what was observed in other earthquakes. For the Wenchuan 2008 earthquake, Li et al.^16^ found that the earthquake-induced landslides volume might effectively offset the addition of coseismic volume. Due to a lower number of landslides with respect to the Wenchuan 2008 earthquake (21,151 for our inventory vs 57,150 in Wenchuan 2008 according to Li et al.^16^) the landslide volume of Nepal 2015 landslides is lower than the Wenchuan 2008 landslide volume, despite similarity in moment magnitude (M_w_). In this analysis, we estimated a volume ranging from 251 (− 15/ + 16) Mm^3^ (method V_AR_river_R1) to 1503 (− 183/ + 210) Mm^3^ (method V_ALL_R2), while the volume of Wenchuan 2008 landslides was estimated as high as 2800 (− 700/ + 900) Mm^3^ (Li et al. 2014). In any case, the total landslide volume associated to the Nepal earthquake fits the empirical landslide volume/earthquake magnitude curves proposed in the literature^2,9,10^ (see Supplementary Figure S4), as also found by Roback et al.^17^.

The overall net volume change is positive for the whole study area, thus demonstrating that the 2015 Nepal earthquake is net constructional for the Nepal Himalayan belt. This is observable considering both the whole area and the sub-areas. Sub-area 1 and sub-area 4 show a greater contribution of landslide volume, essentially due to a higher concentration of landslides.

This positive balance is even larger if considering that most of the material eroded by landsliding remains along the slopes or within the lower-order channel network for long periods, as observed for the Wenchuan earthquake^38,39^. We have tried to roughly estimate the material that can be more easily evacuated from the study area by fluvial transport by considering only landslides that reach the channel network (V_AR_river). This amounts to a total volume ranging between 251 (− 15/ + 16) Mm^3^ (R1) and 582 (− 72/ + 83) Mm^3^ (R2), corresponding to 40% and 77% of V_ALL R1 and V_AR R1, and 39% and 88% of V_ALL R2 and V_AR R2, respectively. A more detailed analysis is recommended to define the channel network's capability to remove such material.

The second hypothesis of the paper is that the uncertainty related to either the landslide volume or the vertical coseismic displacement is very high.

Regarding the landslide volume calculation, we identified and analysed four sources of uncertainty; the first and second related to the choice of the empirical equations and the uncertainty about their parameters, respectively; the third related to the landslide inventory; and the latter related to the approach adopted for the assessment of elongated landslide volume.

As already reported in the literature^6,8,17^ the choice of the empirical area/volume equation introduces a large source of uncertainty. In order to test this uncertainty, we adopted two different equations from Larsen et al. (2010), the first for all the landslides (R1) and the second for mixed-debris and rock slides in Himalaya (R2)^36^. For the method V_ALL the two equations return a mean value of landslide volumes of 628 Mm^3^ and 1503 Mm^3^ for R1 and R2, respectively (Fig. 4a,b). This difference is due to the fact that the Larsen et al.’s^36^ equation for mixed landslides in the Himalaya predicts landslides deeper with respect to the global dataset, especially for larger landslides, with large values for both the α and γ scaling parameters of the power-law equation. The sensitivity of the choice of empirical equation in volume estimation is lower for V_AR and V_AR_river due to the smaller size of the scar areas with respect to the whole landslide area.

The uncertainty about the parameters of the empirical equations is reported in Larsen et al.^36^ and derives from the dispersion of the empirical data used by the authors to obtain the equations. By propagating this uncertainty in the calculation of the landslide volume erosion for the entire inventory we obtained a range of variation ranging from a minimum of 4.9% (V_AR_R1, where the maximum variation is 16 Mm^3^ with a mean value of 324 Mm^3^) to a maximum of 14.3% (V_AR_river _R2, where the maximum variation is 83 Mm^3^ with a mean value of 582 Mm^3^).

The third source of uncertainty lies in the quality and completeness of the landslide inventory. To test and quantify this uncertainty, we calculated the landslide volume from different inventories by applying the method V_ALL R1. First of all, we used the online available landslide inventories for the 2015 Nepal earthquake^17,27,29^. The landslide volumes calculated with these inventories show dramatic differences, with values varying from 6 Mm^3^ for Zhang et al.^27^ to 209 Mm^3^ for Gnyawali and Adhikari^29^ to 343 Mm^3^ for Roback et al.^17^, and to 628 Mm^3^ for our inventory. The differences with Zhang et al.^27^ and Gnyawali and Adhikari^29^ are mainly due to a different number of landslides in the inventories, especially for Zhang et al.^27^ which reports only 2645 landslides. The difference with Roback et al.^17^ is due to the size of the landslides, which is slightly larger in our inventory (see Supplementary Figure S5). The integration of Roback et al.^17^ landslides in our inventory in unmapped area, show smaller differences in total landslide volumes due to a limited number of unmapped landslides, suggesting that they are both almost complete.

A further comparison with volumes reported in Roback et al.^17^, ranging from 130 to 1270 Mm^3^, and Xu et al.^40^, equal to 964 Mm^3^, confirms the strong controls of inventories and empirical relationships in the assessment of the landslide volume.

The fourth source of uncertainty lies in the approach used for the volume computation of landslides with high mobility and aspect ratio. These landslides are usually very shallow, with mass wasting occurring in the scar area due to sliding or toppling that is successively fed by to erosion along the slope. Since the volume is typically calculated by empirical equations starting from volume–area relationships, the choice of the appropriate area or relationship is fundamental.

If the volume is calculated by using the entire landslide area (i.e. V_ALL), the empirical equation would yield a depth that is too large for shallow elongated landslides, resulting in a strong overestimation of the volumes. This may have partially occurred in Parker et al.^6,41^ leading to possible incorrect estimate of the landslide volume.

On the other side, volumes calculated by considering only the scar area would neglect (or account for it only implicitly) the entrainment of material occurring along the runout zone, which may be considerable for long-runout landslides such as debris flows^42,43^.

For this reason, we tested in this paper a hybrid approach for long-runout landslides, consisting of decoupling the volume of the scar source (V_AR and V_AR_river) and the contribution of erosion along the runout zone. We believe this approach to be more realistic in the description of the mechanisms responsible for mass wasting of long runout landslides, such as debris flows, although there is a strong uncertainty on the erosion depth, which depends on local geologic and soil conditions. However, as shown in Fig. 4a,b, the volumes calculated by decoupling the initial scar and the transport erosion are always much lower with respect to the V_ALL method, also considering different erosion thicknesses along the runout (from 0.5 to 3 m). For instance, by using equation R1, the volume calculated by decoupling scar area and runout ranges from 283 to 490 Mm^3^ for V_AR method, versus 628 Mm^3^ for the V_ALL method. This difference is even larger for R2, due to the effects of the different scaling parameters. For a more accurate estimate of the volume, a detailed analysis of the soil thickness would be necessary.

Regarding the vertical coseismic displacement, we adopted for the LOS displacement a RMSE of 54 mm according to Wang and Fialko^37^ to define the uncertainty on the volume estimation. This uncertainty is able to control the uncertainty on the final net volume change estimate being even two orders of magnitude higher than the uncertainty defined for the landslide volumes.

In addition, we analysed the degree of uncertainty associated to the choice of the InSAR dataset, obtaining a difference of about 20% between the ALOS-2 and Sentinel-1, with net volume change values of 1417 Mm^3^ and 1841 Mm^3^, respectively. To assess the reliability of the calculated vertical coseismic displacement, we also compared these InSAR-derived data with vertical GPS records^22^. As shown in Supplementary Figure S3, both ALOS-2 and Sentinel-1 show a very good agreement with GPS data. This validates the use of ALOS-2 data for the characterization of the vertical coseismic displacement in the study area.

## Conclusion

The main findings of the paper are:

- the volume balance of the 2015 Nepal earthquake is strongly dominated by tectonic displacement, with landside erosion being only a small fraction of the overall balance.
- The uncertainties in the calculation of landslide volumes are manifold, and very high. We identified and quantified uncertainties related to the choice of the empirical equations and the uncertainty about their parameters, the completeness and quality landslide inventory, the approach adopted for the assessment of elongated landslide volume, and the InSAR displacement data. In fact, these uncertainties can be as high as the volume itself.
- The different landslide types may require different approaches for the calculation of the volume. We argue in this paper that the classical approach based on empirical area/volume equation may be unsuitable for elongated landslides. We tested a new method decoupling the scar area and the runout components of the volume. With this method, we demonstrate that, although the results are very uncertain due to the difficulty to estimate the erosion thickness, the classical approach tends to overestimate the volumes for such landslides.

## Methodology

### Landslide mapping

**Figure 6:**
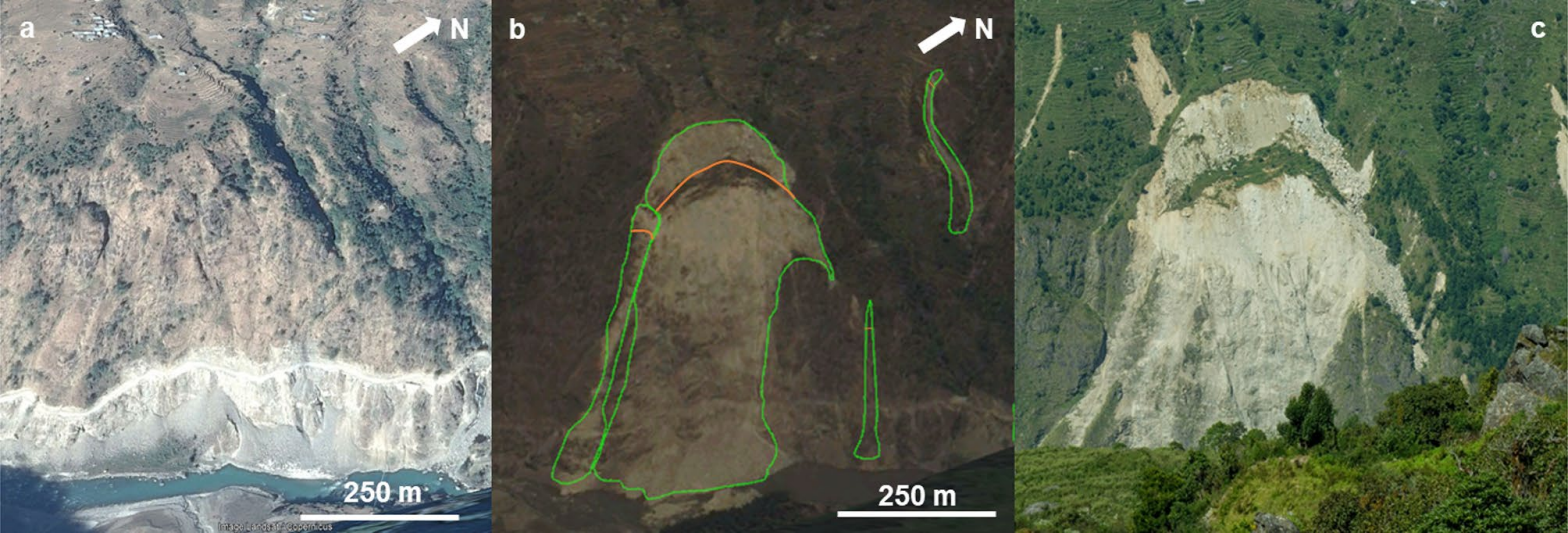
Example of coseismic landslides mapped as polygons in Rasuwa District. (**a**) Satellite image from Google Earth with pre-seismic condition (date 12/12/2014, map data: Google, 2020 CNES / Airbus, https://earth.google.com/web/). (**b**) Satellite image from Google Earth, with an oblique view of the slope (date 5/25/2015, map data: Google, 2020 Maxar Technologies, https://earth.google.com/web/). Orange line delineates the scar sources. (**c**) Field photograph of the rotational slide made during the field activity carried out in October 2015 (photo by Frattini Paolo). The landslide on the west of the scar was not mapped in (**b**) because it was a post-monsoon landslide. (The figure was generated by using Microsoft PowerPoint 2013, http://www.microsoft.com/).

The landslide inventory was prepared through manual mapping of landslide polygons based on multi-temporal Google Earth and Google Crisis Imagery from May 2, 2015 to June 6, 2015. Google Crisis imagery included DigitalGlobe WorldView-2, WorldView-3, and Airbus Pleiades imagery. Also helicopter-based videos recorded by USGS^44^ and OM (Operation Mobilisation) Nepal were used. The inventory was prepared at 1:2000 scale to avoid undersampling of small landslides, especially away from the seismic source. Smaller landslides, in fact, are not systematically accessible and could be easily censored or amalgamated, even with recent imagery^33,45^. Due to the presence of clouds or low quality images about 1500 km^2^ out of 26,240 km^2^ remained unmapped (Fig. 1). Field checks of a small part of the landslide inventory were carried out in October 2015 in the Rasuwa and Sindhupalchok districts and a subsequent validation based on the photos taken on the field was carried out (Fig. 6b,c). About 5% of the landslides in the two districts were checked on site (~ 500 landslides). During the field activities, information provided by locals allowed to recognize and ignore the monsoon-induced landslides. The size frequency distribution of the inventory is reported in Supplementary Figure S5. To manage manual mapping consistency of interpretation and reduce possible errors, the inventory was entirely mapped by a single geomorphologist, but crosschecked with other expert mappers and by using all the available data.

### Landslide scar area

In order to extract the scar size (i.e. the source area of the detachment) from the landslide size, a subset of 1500 landslides located in the central part of the inventory area (Fig. 1) was analysed for detailed mapping of the scar based on high-resolution imagery available in Google Earth (~ 7% of the inventory, Fig. 1) selected on the basis of the image quality and to provide a representative population of the different types. For such representative subset, both the scar ratio (i.e. the slope of the best-fitting linear regression between scar area and total landslide area) and the aspect ratio (AR, the ratio between the landslide length and width) were calculated.

The shape of the landslide was characterized by the aspect ratio (AR), which is defined as the ratio between the landslide length (L) and the landslide width (W) and computed by enveloping the landslide polygons with bounding rectangles^46,47^. Based on this aspect ratio, the landslides were classified into six classes (< 2, 2–4, 4–6, 6–8, 8–10, and > 10). For each class, a different linear function relating the total landslide area with the scar area was defined (Fig. 2; Table 1). These equations were applied to all the landslide inside the inventory to assess the landslides scar areas.

### Landslide volume and erosion

**Figure 7:**
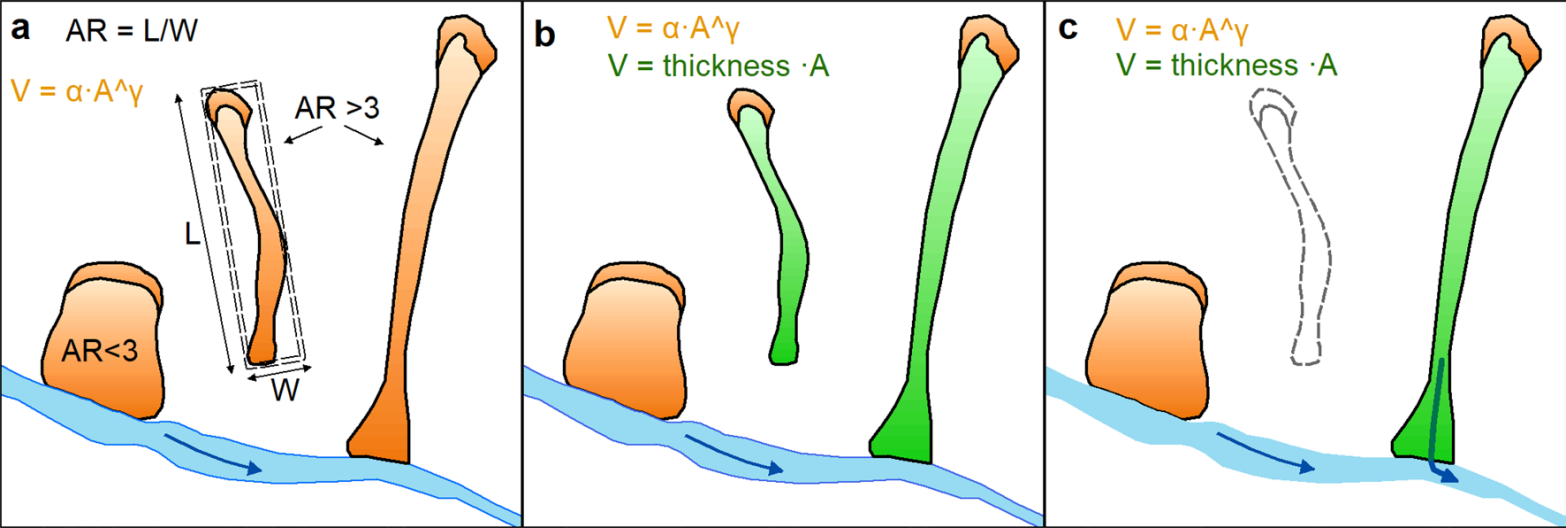
Schematic representation of the three methods for volume calculation. (**a**) V_ALL: Larsen et al.^36^ relationship was applied to the entire landslide area with the two parameterizations (R1 and R2)^36^. (**b**) V_AR: Larsen et al.^36^ for landslides with AR < 3, and for scar area of landslide with AR > 3; for the runout of landslides with AR > 3, the volume was defined as the area multiplied for the thickness. (**c**) The methodologies were applied to landslides that overlap the river network with the same method of “b” case”.

To analyse the amount of landslide material removed by the earthquake, the area that corresponds to the envelope of the earthquake-induced landslide inventory was divided into a lattice (235 cells) with a dimension of 10 × 10 km. For each cell of the lattice, three different values of volume eroded by landslides were calculated (Fig. 7):

- V_ALL: the volume was defined for all the landslides inside the inventory by using the empirical area/volume relationship by Larsen et al.^36^ considering the total area of each landslide (A_i_), without any distinction between scar and runout areas^36^ (Fig. 7a):
  1 $${V}_{tot}=\sum_{1}^{n}\alpha \cdot {A}_{i}^{\gamma }$$
  where V_tot_ is the total volume for all landslides, n is the number of landslides and the scaling parameters α and γ are constants that vary as a function of the geological and geomorphological settings and the hillslope process. The constants α and γ used in this work are those proposed by Larsen et al.^36^ as a global relationship for all kinds of landslide (R1) and a relationship for mixed bedrock and soil landslides in the Himalaya (R2). Although the empirical area/volume relationship proposed by Lacroix^28^ refers to the 2015 Nepal landslides, it is limited to Langtang valley and was not used because we believe it may be too local scale to be representative for the entire dataset.
- V_AR: the inventory was divided into elongated (AR > 3) and non-elongated (AR ≤ 3) landslides. For non-elongated landslides, the area/volume relationships R1 and R2 were applied to the total area (as for V_ALL). For elongated landslides, the volume was calculated by decoupling the scar from the erosion along the runout. The scar contribution was calculated with area/volume relationships (R1 and R2) applied to the scar area only (Fig. 7b), while the contribution of erosion along the runout was calculated by assuming an erosion thickness of 0.5, 1 and 3 m. This range of values was adopted based on field observations and assuming an erosion depth corresponding to the soil thickness^48–50^.
- V_AR_river: the volume was defined only for landslides that overlap the channel network (i.e. 23% out of total inventory) by using the same approach V_AR (Fig. 7c). To be conservative, a raster-based criterion was adopted to identify all landslides that overlap directly with the channel network, defined by calculating the flow accumulation parameters from the Shuttle Radar Topographic Mission (SRTM) GDEM (Global Digital Elevation Model) with a resolution of 3 arc second (about 90 m resolution, SRTM-3)^51,52^. A drainage area of ~ 0.48 km^2^ (~ 60 contributing cells), as proposed by Roback et al.^17^ was selected.

The uncertainty on the volume estimation was assessed with mean values ± 16th and 84th percentiles of 10,000 times Monte Carlo sampling for α and γ parameters.

Starting from these volumes and considering the area of each cell, the mean erosion (E_cell_) is calculated as (Fig. 3):

2 $${E}_{cell}={V}_{tot}/{A}_{cell}$$

where A_cell_ is the lattice cell area (10 km × 10 km) and V_tot_ is the total volume of all the landslides located in each cell of the lattice.

### Mean vertical coseismic displacement

Inside each cell of the lattice, the mean vertical coseismic displacement (VCD) due to tectonic displacement is given by:

3 $${VCD}_{cell}=\frac{\sum_{x=1}^{n}{VCD}_{x}{A}_{x}}{{A}_{cell}}$$

where the numerator corresponds to the coseismic volume change; A_x_ is the ALOS-2 grid-cell area (88 × 88 m); VCD_x_ is the vertical displacement in each ALOS-2 cell; n is the total number of cells. This value corresponds to the average of the VCD_x_ within the lattice cells. VCD_x_ represents the vertical displacement only, and was calculated by dividing the LOS displacement by the cosine of the incidence angle, that ranges from 27° to 49° from West to East for the five subswaths^19^. This simplified calculation was possible because the LOS vector resulting from the descending geometry of ALOS-2 is nearly parallel to the strike of the Main Frontal Thrust, thus reflecting only the vertical displacement.

### Comparison among different inventories

The method V_ALL R1 was applied to three published inventories available online (https://www.sciencebase.gov/catalog/item/587352ebe4b0a829a31e309a, see Supplementary Figure S1):

- Zhang et al.^27^ (2645 landslides).
- Gnyawali and Adhikari ^29^ (17,638 landslides).
- Roback et al. ^17^ (24,915 landslides).
- integration in our inventory of landslides in Roback et al.^17^ that are located in areas (1500 km^2^ out of 26,240 km^2^) unmapped due to the presence of clouds or a very high distortion of aerial images (21,654 landslides).

## Supplementary Information

Supplementary Information
