# Supplementary material for: Role of landslides on the volume balance of the Nepal 2015 earthquake sequence
Source: Sci Rep. 2021 Feb 9;11:3434. doi: 10.1038/s41598-021-83037-y (PMC7873231; doi:10.1038/s41598-021-83037-y)
Supplement: Supplementary file 1 — Supplementary Information [file 41598_2021_83037_MOESM1_ESM.docx]

# Supplementary information

Role of landslides on the volume balance of the Nepal 2015 Earthquake sequence

A. Valagussa, P. Frattini, E. Valbuzzi & G.B. Crosta

Università degli Studi di Milano-Bicocca, Dept. Earth and Environmental Sciences, Milano, Italy

[andrea.valagussa@unimib.it](mailto:correo_electrónico@autor.correspondiente)

## Supplementary Figure


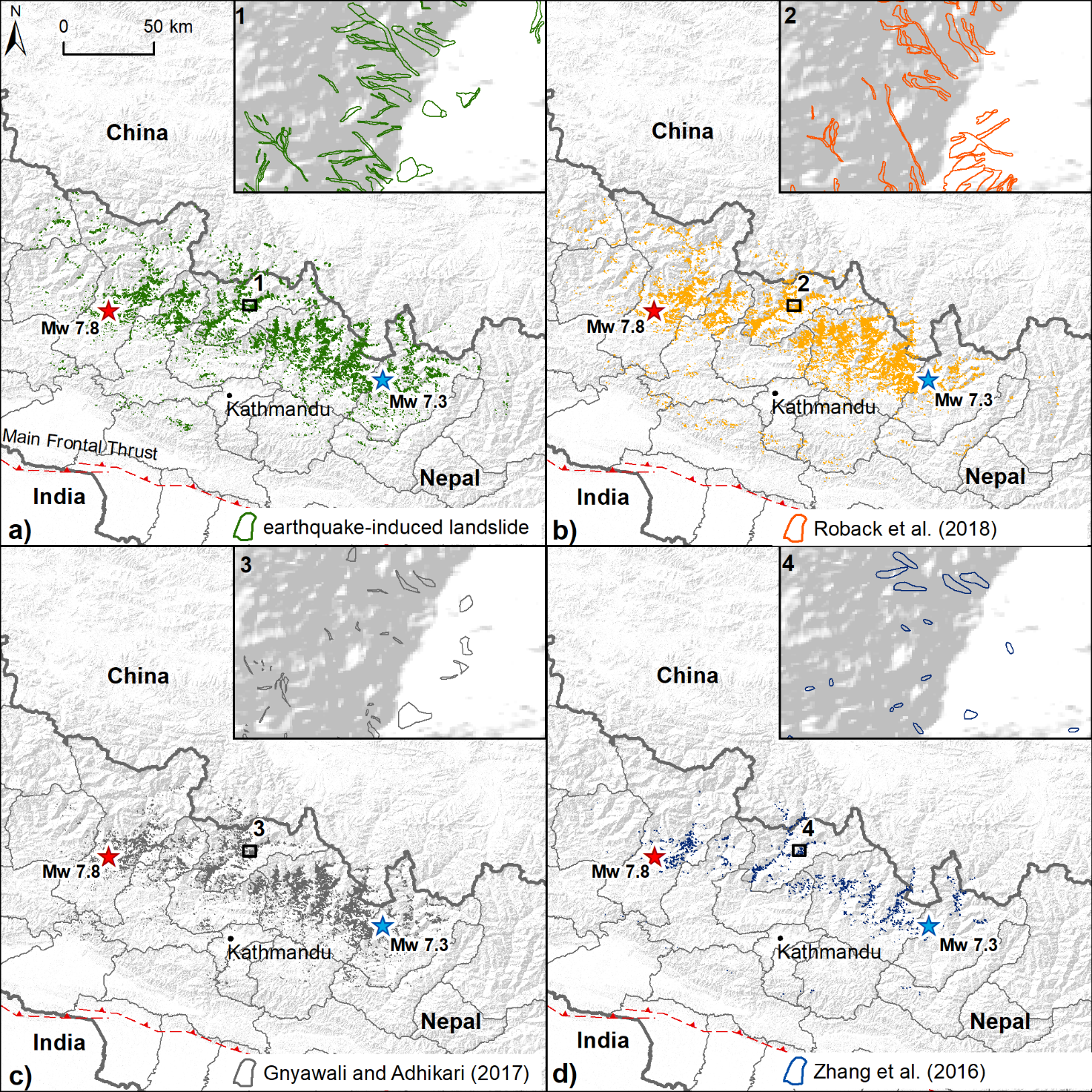


Supplementary Figure S1: Comparison of the landslide inventories available and analysed in the present work: a) our inventory with 21,151 landslides; b) Roback et al.^17^ with 24,915 landslides; c) Gnyawali and Adhikari^29^ with 17,628 landslides; c) Zhang et al.^27^ with 2,645 landslides. The areas covered by the inventories are different as the number of mapped landslides. Insets 1, 2, 3, and 4 show example of differences in mapped landslides among the inventories. (The maps were generated by using ArcGIS 10.3.1, <http://www.esri.com/>).


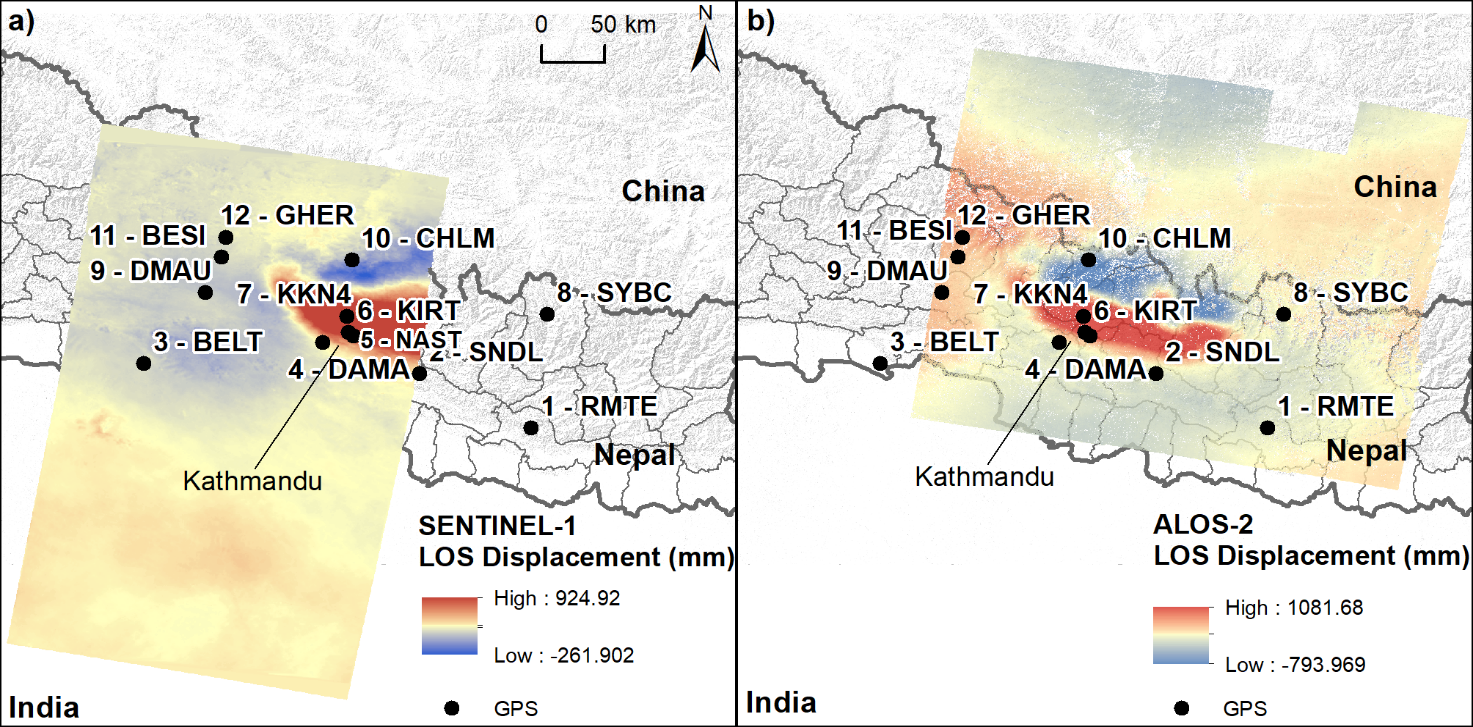


Supplementary Figure S2: Maps of the LOS displacement (mm) for a) SENTINEL-1, coseismic interferogram April 17 - April 29 2015, and b) ALOS-2, interferogram spanning over the main shock and the main aftershock, 22 February -17 May 2015. Locations of GPS stations are reported for reference (see supplementary figure S3b). Note different colour scale ranges. (The maps were generated by using ArcGIS 10.3.1, http://www.esri.com/).





Supplementary Figure S3: a) comparison between the vertical coseismic displacement for the ALOS-2 and Sentinel-1 satellites with respect the coseismic offset of the GPS stations inside the affected area^22^. Number refer to the GPS stations shown in b) (The map was generated by using ArcGIS 10.3.1, http://www.esri.com/). As for ALOS-2 the LOS displacement captured by Sentinel-1 was converted into the vertical coseismic displacement by dividing the LOS displacement by the cosine of the incidence angle, that ranges from 30.9 degree and 45.9 degree from West to East as reported by the ESA website

(http://insarap.org/data/nepal/S1_Nepal_Coseismic_20150417_20150429_GEOMETRY.txt).

The analysis shows a good agreement between the GPS data and the vertical coseismic displacement (VCD) derived from the LOS displacement for the both the satellites.


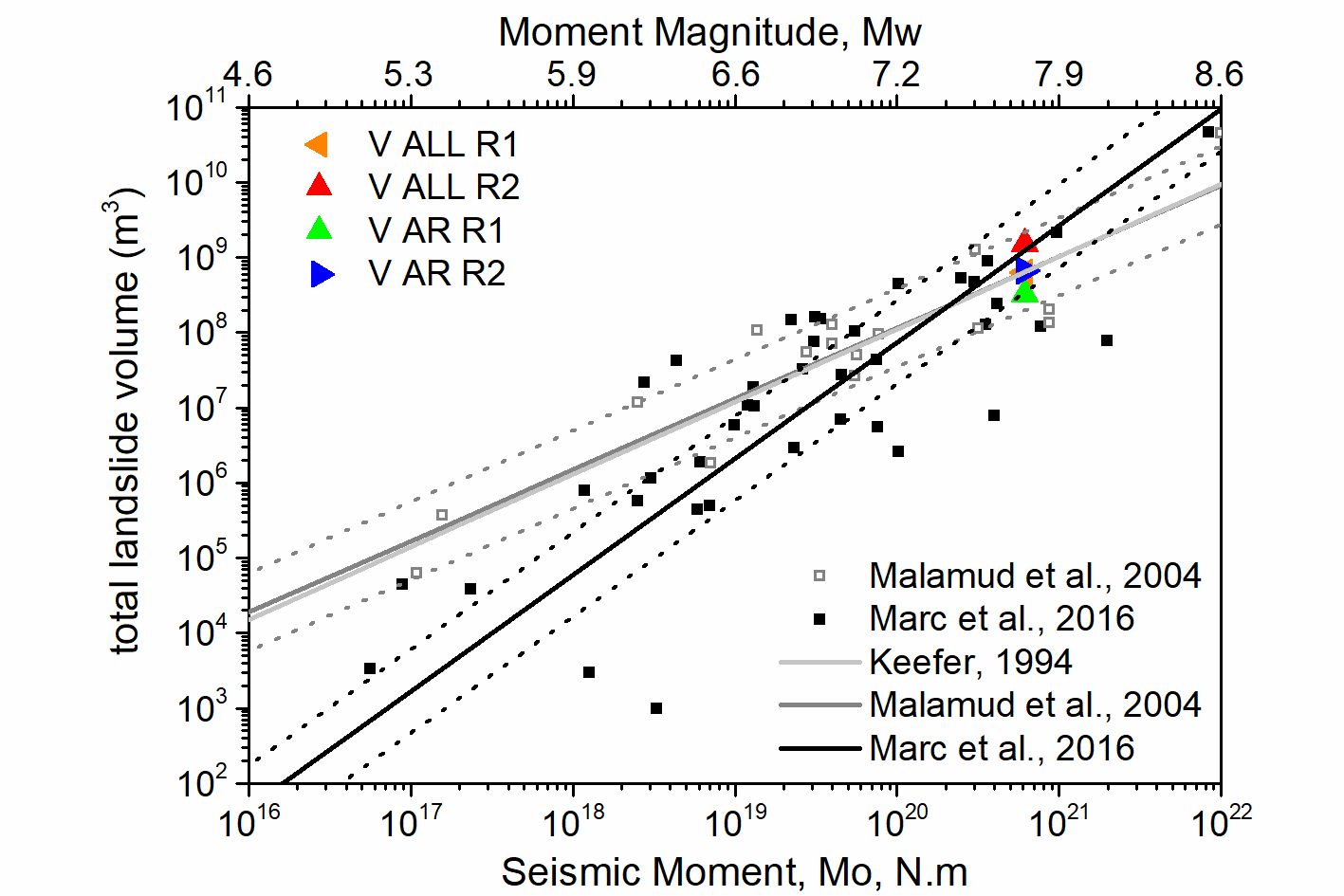


Supplementary Figure S4: Relationship between earthquakes seismic moment M_0_ and moment magnitude Mw and the total landslide volume of earthquake-induced landslides. Solid and dashed lines are the fitting lines proposed by Keefer^2^, Malamud et al.^9^, and Marc et al.^10^ based on a dataset of 15, 16, 40 landslide events, respectively (black and grey empty square see Malamud et al.^9^, and Marc et al.^10^ for earthquakes references). The total volumes calculated for the Nepal 2015 earthquake (triangles) are calculated by using methods V_ALL and V_AR with R1 (global relationship for all landslides) and R2 (relationship for Himalayan landslides), using Larsen et al.^36^ relationship.

*
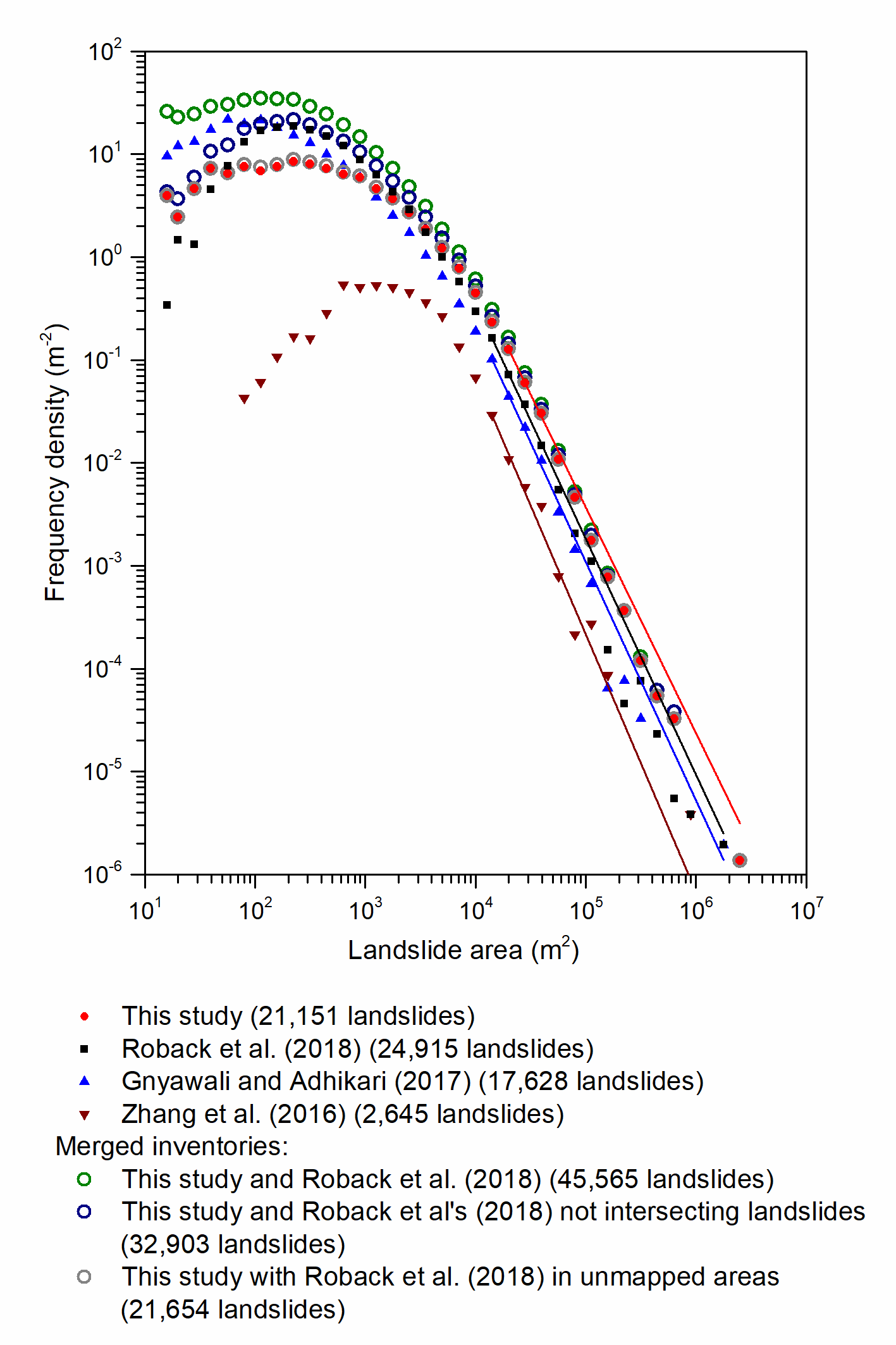
*

Supplementary Figure S5: Magnitude frequency curves for all the earthquake-induced landslide inventories considered. In general, it can be observed that for landslide sizes larger than 10^4^ m^2^ our inventory shows a frequency density (m^-2^) higher than those already available in the literature. The integration of Roback et al’s inventory^17^ with our inventory defines an increase in frequency density (m^-2^) for landslides smaller lower than 10^4^ m^2^. A power-law behaviour of the MF curves above a certain size threshold is observed. For the power-law fitting of the right tail of the distribution, the least-squares algorithm of Levenberg–Marquardt was applied. The scaling parameters are -2.34 for the inventory proposed by Gnyawali and Adhikari^29^; -2.29 for Roback et al.^17^; -2.19 for our inventory and -2.18 for Zhang et al.^27^. This parameter describes the relative proportion of larger and smaller landslides in the inventory. Lower the parameter, higher the proportion of larger landslides. Zhang et al^27^ and our inventory show the higher proportion of larger landslides. The reason for Zhang et al. (2016) value could be related to the limited number of landslides.

## Supplementary Table

Table S1. Comparison between linear functions obtained in this study for the scar area vs landslide area relationship based on the 1,500 selected landslides (see Fig.1), and the function developed by selecting the landslides from Roback et al.^17^ in the same area of our analysis. Linear functions describe the relationships between the scar area (A_s_) and the total landslide area (A), for different classes of aspect ratio (AR). Roback et al’s landslide^17^ scar areas are larger than our scar areas, with the exception of AR between 2 and 4. The difference between the two values reaches a maximum for an AR between 6 and 8 where for Roback et al.^17^ a percentage of 32.7% of the entire landslide body with respect our 6.2%. In general, the percentages from Roback et al.^17^ are larger, especially for long runout landslides.

|  | Our Inventory | | Roback et al. (2018) | |
| --- | --- | --- | --- | --- |
| AR | Fitting function | Landslide scar area (As,%) | Fitting function | Landslide scar area (As,%) |
| < 2 | As = 0.093A | 9.3% | As = 0.171A | 17.1% |
| 2 - 4 | As = 0.102A | 10.2% | As = 0.094A | 9.4% |
| 4 - 6 | As = 0.065A | 6.5% | As = 0.160A | 16.0% |
| 6 - 8 | As = 0.062A | 6.2% | As = 0.327A | 32.7% |
| 8 - 10 | As = 0.046A | 4.6% | As = 0.137A | 13.7% |
| > 10 | As = 0.040A | 4.0% | As = 0.188A | 18.8% |
